# Synthesis of Silver Nanoparticle/Multi-Walled Carbon Nanotube Composites and Their Application in Electronic Pastes

**DOI:** 10.3390/nano15030152

**Published:** 2025-01-21

**Authors:** Zizhen Wang, Ming Zhou, Baoying Lu, Duo Zhang, Hui He

**Affiliations:** 1 School of Mechanical and Automotive Engineering, Guangxi University of Science and Technology, Liuzhou 545006, China; 2 Guangxi Jianxing Guangyin New Material Technology Co., Ltd., Nanning 530024, China; 3 Guangxi Earthmoving Machinery Collaborative Innovation Center, Liuzhou 545006, China

**Keywords:** silver nanoparticles, multi-walled carbon nanotubes, electronic paste, thermal conductivity

## Abstract

Silver nanoparticle-coated multi-walled carbon nanotube (Ag/MWNT) composites were prepared using a chemical plating method that effectively controls the overgrowth of silver nanoparticles, ensuring uniform particle size. Functionalization of the carbon nanotube surface with numerous functional groups facilitates the binding of silver ions to multi-walled carbon nanotubes (MWNTs). This process results in Ag/MWNT composites with a uniform distribution of silver nanoparticles, prepared through reduction via the silver mirror reaction. The impact of dispersants and reducing agents on the silver coating of carbon nanotubes was studied. The results revealed the formation of negatively charged functional groups (-COOH, -OH, -C=O, and -NH2) on the nanotube surface. These groups acted as nucleation sites for the formation of silver nanoparticles. These groups acted as nucleation sites for the formation of silver nanoparticles. Simultaneously, the Ag/MWNT composites demonstrated effective dispersion within the matrix, improving the electrical conductivity of the electronic paste by 32.1% and 33.1%. This improvement was attributed to the forming of a conductive pathway within the silver-modified composite. Ag/MWNT composites within the paste system improved interfacial contact between fillers and the matrix, enhancing their potential applications in thermal interface materials.

## 1. Introduction

With the growing demand for high-speed electronic products, integrated circuits, particularly chips, generate increasing amounts of heat. This requires the use of heat sink materials to enhance heat dissipation and advances in thermal interface and management technologies [1,2,3,4,5,6,7,8,9]. Though offering high thermal conductivity, gold-tin solder poses a risk of chip damage during encapsulation due to its elevated temperature [10,11,12,13]. Often used in thermal interface materials, electronic pastes meet interconnect packaging and electrical conduction needs. However, their thermal conductivity of around 0.7 W/m K is inadequate, highlighting the need for new materials and methods to address the limitations of low-temperature curing pastes. Recently, high-performance thermal interface materials have reached thermal conductivities of 20–50 W/m K, with silver-based materials exceeding 50 W/m K. These materials are well-suited for high-power, high-density electronic components and are now a major research focus in both industry and academia [14,15,16].

Multi-walled carbon nanotubes (MWCNTs) offer superior properties, including a high specific surface area, increased tensile strength and toughness, and exceptional thermal (3000 W/m K) and electrical conductivity, outperforming metallic and ceramic thermally conductive fillers [17,18,19,20]. Depositing metallic silver onto MWCNTs can improve thermal and electrical conductivity [21,22,23]. Additionally, Ag nanoparticle (NP)-modified MWCNTs show a synergistic effect, improving electrical and thermal conductivity and material toughness [24,25,26]. Hemant Pal et al. [27] used molecular-level mixing to prepare MWNT-reinforced silver matrix composites, studying the impact of functionalized MWNTs on thermal conductivity. Their findings showed that functionalized MWNTs increased pure silver’s thermal conductivity from 430 W/m K to 530 W/m K, highlighting the potential of these composites. Yanchao Li et al. [28] used chemical and ball milling methods to attach Ag nanoparticles to MWNT surfaces, enhancing thermal conductivity from 0.65 W/m K to 0.75 W/m K. However, they did not analyze thermogravimetric or morphological changes in the Ag/MWCNT composites, and the ball milling process may introduce impurities, lowering purity. Yunkai Wang et al. [29] first prepared high-loading Sn-MWCNT composites using a chemical replacement method, then replaced tin with silver ions to create Ag-MWCNT composites. The silver nanoparticles were uniformly distributed on the nanotube surface, significantly enhancing thermal conductivity. However, tin mainly exists as stable +4 ions during the replacement reaction, rapidly increasing silver content and causing nanoparticle aggregation. Jun Natsuki et al. [30] developed a one-step green method to attach Ag nanoparticles to MWCNTs, obtaining uniform 5 nm Ag nanoparticles on the surface through simple separation. This approach advances the use of Ag/MWNT composites in electronic pastes. Daewoo Suh et al. [31] discovered that Ag/MWNT thermal interface materials performed optimally with a thermal conductivity of 160 W/m K. The nano-silver on the nanotube surface enhances thermal conductivity by creating an efficient thermal pathway between silver flakes, improving carrier concentration and mobility. Behnam Meschi Amoli et al. [32] examined the use of silver nanoparticles in functional materials, highlighting the impact of surface chemistry on their properties. They found that short-chain-modified silver nanoparticles are conductive, while long chains are not. When used in conductive adhesives, short-chain nanoparticles significantly improve conductivity. Behnam Meschi Amoli et al. [33] investigated the effects of different polymers and conductive fillers—such as silver nanoparticles, micrometer-sized silver flakes, carbon nanotubes, and graphene—on electrically conductive adhesives (ECAs). They suggested new methods for enhancing conductivity and proposed future research directions, emphasizing the need to consider the economic feasibility of these materials in future practical applications.

This study investigates the preparation of functionalized MWNTs using a chemical method, followed by the fabrication of Ag/MWNT composites through chemical plating. This process enhances Ag NP dispersion while maintaining composite purity. The composites exhibit a synergistic effect by combining the benefits of both MWNTs and Ag NPs. The study further explores the potential of MWNTs as a matrix material and filler in electronic pastes, aiming to enhance their electrical and thermal properties. The findings present a method for preparing Ag/MWNT composites and provide valuable insights for their use in high-conductivity electronic pastes.

## 2. Materials and Methods

### 2.1. Chemicals

All experimental reagents used in this thesis are listed in Table 1, and all reagents can be used without further purification.

### 2.2. Methods

Synthesis of Ag/MWNT: The initial step in the preparation of Ag/MWNT involved the amino-functionalization of MWNTs with nitric acid (HNO_3_) at a temperature of 105 °C. Subsequently, the excess HNO_3_ was neutralized using NaOH. The amount of carboxyl groups present on the surface of the MWNTs was determined by measuring the consumption of NaOH. It was determined that approximately 30% of the nitric acid was consumed. The initial step in the surface pretreatment of MWNTs involved acidification. Subsequently, the mixed solution of MWNTs was washed with deionized water and filtered using a membrane. This process was repeated multiple times to ensure the isolation of pure functionalized MWNTs. Following this, the functionalized MWNTs were added into sodium dodecyl sulfate (SDS) solution and sodium dodecylbenzene sulfonate (SDBS) solution, respectively, and dispersed in an ultrasonic bath under an ice bath. The dispersed suspension was added to the AgNO_3_ solution and dispersed for approximately two hours. The dispersed suspension was mixed with MWNTs, sodium sulfate, and sodium benzenesulfonate. The AgNO_3_ solution was then introduced to the dispersed suspension and stirred in a magnetic stirrer for approximately 6 h. Subsequently, the reducing agent ascorbic acid solution (0.1 mol/L) was added to the system drop by drop at 10 mL per minute at room temperature. At the same time, the magnetic stirring was carried out. The stirring was continued at room temperature for approximately 0.5 h. Finally, Ag/MWNT composites were prepared by reducing Ag NPs from the MWNTs with functionalized surfaces via a silver-mirror reaction. MWNT composites were obtained by centrifugal collection with ethanol washing several times after standing overnight.

Preparation of thermally conductive adhesives from Ag/MWNT-filled electronic pastes: The experimental dosage of the electron was 20 g, including the amount of silver powder used. In this experiment, the experimental dosage of electrons was set at 20 g, and the content of silver powder was set at 60 wt.%. Five samples were prepared by adding silver–carbon composite materials of different qualities. The steps involved in slurry preparation are as follows: Initially, the organic carrier and silver powder were pre-mixed into a slurry using a mixer. Subsequently, silver-modified carbon nanotubes were added and dispersed using a three-roller mill. Finally, a modified electronic paste was obtained through depressurization and defoaming. The viscosity test, performed by hand coating the paste filled with 10 mm × 10 mm mold, involved the use of a mold for a copper plate with a 10.2 mm × 10.2 mm hole punched out after the sticking of heat-resistant tape around the bottom of the hole, which was sealed with PET film. The samples were then placed in an oven at 200 °C for 1.5 h. After cooling to room temperature, the samples were removed, and the cross-sectional morphology of the flatness of the sintered sample was observed. The sintered samples were stored in liquid nitrogen for one hour after cooling, and the heat transfer between the samples and the PET substrate was evaluated using a HotDisk (TPS2500) (Hot Disk Instruments, Gothenburg, Sweden). The electrical resistance of the material cubes was measured with a Keithley 2400 four-probe meter to assess the efficacy of electrically conductive adhesives filled with electronic pastes composed of Ag/MWNTs.

### 2.3. Characterization

The X-ray powder diffractometer used in this study was a D8AVANCE model manufactured by Bruker, Germany. This instrument was utilized to analyze the structural alterations of MWNTs under various functional conditions, the growth crystal planes of Ag NPs, and the bonding mode of MWNTs and Ag NPs. The test conditions were as follows: the X-ray source was a Cu target, and the wavelength of the Kα rays was measured to be 1.5406 Å. The monochromator was a graphite type, and the operational voltage and current were set to 40 kV and 300 mA, respectively. The step size was set to 0.01°, and the scanning range was set to 5° ≤ 2θ ≤ 80°. A specific surface area tester (model 3H-2000PS1, manufactured by Bestech Technologies, Inc., Shanghai, China) was utilized to assess the alterations in the specific surface area of MWNTs and Ag/MWNTs. The pretreatment temperature for the specific surface area tester was set at 200 °C. The transmission electron microscope (TEM) utilized in the experiment is the JEM-2100 transmission electron microscope, which a Japanese company produced (JEOL Ltd., Tokyo, Japan). The instrument was used to observe the morphology, size, and dispersion of the MWNT and Ag/MWNT samples at an accelerating voltage of 100 kV. Additionally, it was used to examine the surface state of the Ag NP coating after the MWNT coating. The structural analysis of electron diffraction SAED (Selected Area Electronic Diffraction) mode can be used for single crystal diffraction, polycrystalline diffraction observation, and analysis. The scanning electron microscope (SEM) employed in this particular experiment segment is the Quanta200 field emission scanning electron microscope manufactured by the American FEI company. The morphology and surface structure of the cured electronic pastes and the interconnections between the Ag/MWNT composites and the silver powders were observed using the instrument at an accelerating voltage of 10 kV. Infrared spectroscopy (IR) was used to detect changes in the class and number of functional groups on the surface of the MWNTs treated with different functionalizations. The IR absorption spectrometer was manufactured by Bruker, Germany, model TENSOR27, with a test wave number range of 400–4000 cm^−1^. Raman spectrum (RS) was utilized in this experiment to analyze the defects of MWNTs and the effect of Ag NPs on MWNTs. The Raman spectrometer used in this study was a Renishaw MKI1000, London, UK, equipped with a 532 nm excitation wavelength. This instrument was utilized to detect the surface integrity of MWNTs in both the original MWNTs and the composite materials by scanning the two characteristic peaks of MWNTs. The primary objective of this study is to verify the structural alterations of MWNT surfaces following chemical plating, with the ultimate goal of determining the structural metal layer plating on the surface of the MWNTs. This is achieved by analyzing the change in the ratio R of the characteristic MWNT, D, and G peaks. The wave number range utilized was from 1000 to 2000 cm^−1^, and the number of integrations performed was five. X-ray photoelectron spectroscopy (XPS) was employed in this experiment segment to examine the elemental composition, functional group content, and distribution in Ag/MWNTs obtained from nitric acid treatment. The X-ray photoelectron spectrometer (XPS) used was a model PHI5500 from Ulvac-Phi, Kanagawa, Japan. The Thermal Gravimetric Analyzer (TGA) was employed to ascertain the precise content of the dispersant, MWNTs, in the composite electrode materials and to assess the heat resistance of the paste following curing. The TGA utilized in this experiment is the STA-409PC from NETZSCH, Selb, Germany, with a temperature range of 20–600 °C. The TGA was utilized to ascertain the precise content of MWNTs in the composite electrode material. The conductivity test was conducted using a printed method by applying 0.20 × 100-mm conductive lines to the 400-mesh steel wire mesh plate. The lines were then cured at 160 °C for 20 min. The resistance value of the material square was measured using a four-probe meter. The four-probe meter is the ST-2258C model produced by Suzhou Jingge Electronics Co., Ltd., Suzhou, China. The film thickness of the lines was gauged using a FISCHER-SCOPE (MMS 3 AM) film thickness meter. Thermal conductivity test: The paste was manually coated into the 10 mm × 10 mm mold (a mold for a copper plate punched out 10.2 mm × 10.2 mm holes in the square around the heat-resistant tape, with the bottom of the hole sealed with PET film). The mold was then filled with paste. The film thickness of the lines was measured using a film thickness meter (FISCHER-SCOPE, MMS 3 AM). Thermal conductivity testing: The paste was manually coated into the 10 mm × 10 mm mold. The mold contained a 10.2 mm × 10.2 mm hole punched from a square of heat-resistant tape. The bottom of the hole was sealed with a piece of PET film. The mold was then placed into an oven for baking and firing. This process formed a cubic block. After cooling, the cross-section morphology of the flat paste molding was examined. After one hour, the paste was cut. The heat transfer capacity between the sample and the PET substrate was tested with a testing instrument (TPS 2500, Hot Disk Instruments, Gothenburg, Sweden). The specific surface area, pore volume, and pore diameter of pristine MWNT, functionalized MWNT, and Ag/MWNT composites were analyzed using a Brunauer–Emmett–Teller (BET) specific surface area analyzer, a 3H-2000PS1 from BEST Technology, Beijing, China.

## 3. Results and Discussion

### 3.1. Performance Analysis of Nitric Acid Treated MWNT

Carboxyl functionalization of MWNTs was performed at 105 °C using a 10% nitric acid solution. NaOH was then used to neutralize excess HNO_3_, and the functional group content on the MWNT surface was determined based on NaOH consumption. The acid consumption was approximately 30%. Acidification is the first step in the surface pretreatment of MWNTs. MWNTs are immersed in a 10% nitric acid solution, which oxidizes the surface, introducing negatively charged functional groups such as -COOH, -OH, -C=O, and -NH2. Electrostatic repulsion between these functional groups improves MWNT dispersion and enhances Ag+ adsorption on the MWNT surface [34]. The functionalized MWNTs were then washed with ethanol and filtered through membrane filtration. This washing and filtration process was repeated several times to obtain pure functionalized MWNTs [35]. Carboxylated MWNTs, combined with the dispersant sodium dodecyl sulfate (SDS), were used to investigate the effect of varying silver salt concentrations by adjusting the silver nitrate solution proportions. This controlled the ratio of silver salts to MWNTs, influencing the particle size of Ag+ NPs on the MWNT surface.

**Figure 1:**
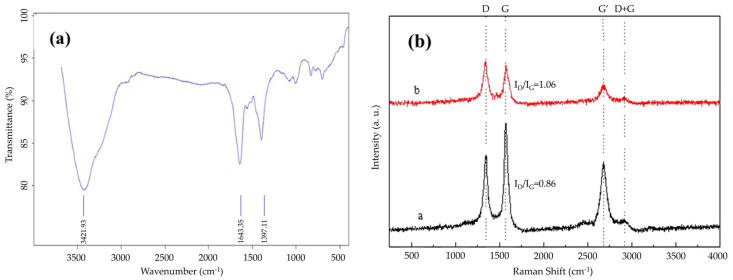
FT-IR spectral analysis (**a**) and Raman spectral analysis (**b**) curve a: original MWNT Raman spectra; curve b: MWNT Raman spectra after functionalization.

As shown in Figure 1a, the vibrational peak at 3360 cm^−1^ corresponds to the C≡C functional group, and the peak at 1740 cm^−1^ is the characteristic -COOH peak. These changes indicate that the surface inertness of the original MWNTs has been altered, enabling them to serve as a nucleation matrix for Ag NPs [36]. Many negatively charged functional groups, such as -COOH, -OH, and -C=O, are present on the Ag NPs’ surface, creating a zeta potential difference in the solution. Electrostatic repulsion between these functional groups improves the dispersion of MWNTs in solution and promotes Ag+ adsorption on the MWNT surface.

Raman spectroscopy is used to characterize the interaction between MWNTs and Ag NPs. The Raman spectra were further analyzed to confirm the coating state of the MWNT surface. Figure 1b shows two distinct peaks in the Raman spectrum. The D peak at 1340–1350 cm^−1^ indicates irregular carbon atoms with dangling bonds at the edges of two-dimensional planes. This peak reflects the disordered structure of the graphite layer. The G peak, located at 1570–1580 cm^−1^, is related to the sp^2^ bond in the graphite layer, representing the vibration of carbon atoms in the two-dimensional hexagonal lattice [37]. This peak reflects the structural characteristics of the original graphite. The intensity ratio of the D (1340–1350 cm^−1^) and G (1570–1580 cm^−1^) peaks in line a of Figure 1b is 0.86, suggesting that the surface structure of the pristine MWNTs remains largely intact.

In contrast, the D to G peak ratio in line b of Figure 1b is higher than in the original MWNTs, indicating an increase in defect density on the surface after acid treatment. The surface of the MWNTs shows a significant presence of negatively charged -COOH, -OH, and -C=O functional groups. The electrostatic repulsion between these groups may affect the electronic structure but preserves the superior surface structure of the MWNTs. Retaining the excellent electrical and thermal properties of MWNTs is beneficial for using silver-modified MWNTs in electronic pastes.

### 3.2. Effect of Different Reducing Agents on the Synthesis of Ag/MWNT

**Figure 2:**
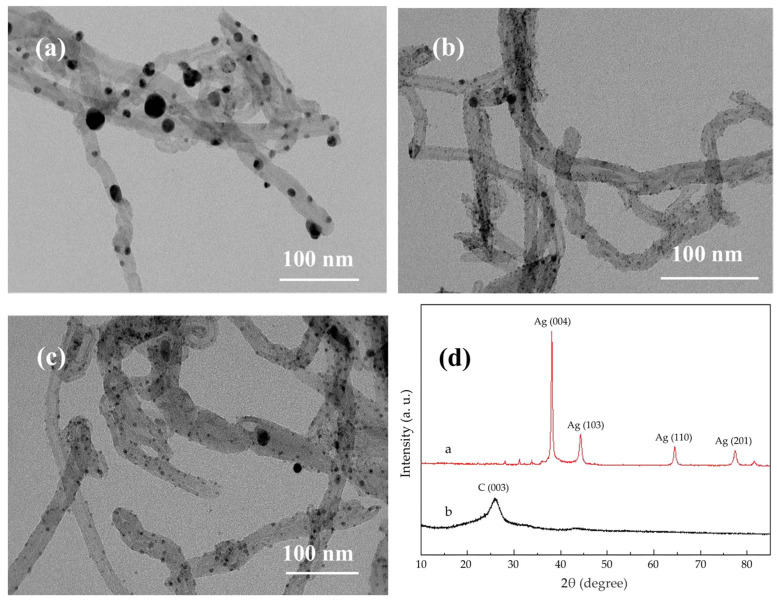
TEM photographs of Ag/MWNT synthesized with different reducing agents. (**a**) Reducing agent is hydrazine hydrate; (**b**) reducing agent is formaldehyde; (**c**) reducing agent is ascorbic acid; (**d**) curve a: XRD image of Ag/MWNT composite; curve b: XRD image of functionalized MWNTs.

The plating state of the Ag/MWNT surface is sensitive to modulation of the reducing agent strength, highlighting the crucial role of the reducing agent solution in maintaining plating integrity [38]. In Figure 2a, MWNT aggregation occurs due to oversized Ag NPs and the entanglement of MWNTs, limiting the effective use of the MWNTs’ large specific surface area. Meanwhile, Ag NPs in the plating solution are rapidly reduced by hydrazine hydrate, leading to their deposition at nucleation sites. These Ag NPs then grow, forming encapsulated MWNTs either laterally or vertically. During chemical plating, the Ag+ concentration gradient in the plating solution causes local Ag+ concentrations to fluctuate, being either excessively high or low. This variation affects the nucleation and growth of Ag NPs on the Ag/MWNT surface, resulting in different observed outcomes. The Ag+ concentration gradient in the plating solution causes localized variations in Ag+ concentration, influencing the nucleation and growth of Ag NPs on the Ag/MWNT surface. The Ag/MWNT surface shows anisotropy in the growth rates of Ag NPs, leading to the formation of large agglomerated Ag NPs. This phenomenon negatively impacts the uniformity of the surface plating layer. These observations align with the morphology of Ag NPs grown on the MWNT surface, as observed through transmission electron microscopy. Figure 2b shows the reduction of Ag NPs on MWNTs using formaldehyde, where fewer Ag NPs are dispersed on the MWNT clusters, leading to less uniform dispersion and lower silver loading. The most effective reduction occurred when using an ascorbic acid reductant solution (Figure 2c), facilitating the reduction of Ag+ onto the MWNT surface. Uniformly adhered Ag/MWNT composites were obtained, demonstrating the successful modification of Ag NPs on the MWNT surface. Ascorbic acid, a weak reducing agent, slows the deposition reaction. Curve a in Figure 2d shows that the Ag NPs on the MWNT surface are fully crystallized, with sharp peaks in the silver-modified carbon nano-XRD pattern. As shown in Figure 2d, curve b presents the XRD image of functionalized MWNTs, displaying characteristic peaks at the C(003) crystal plane, consistent with those reported in the literature [28].

### 3.3. Effect of Different Dispersants on Ag/MWNT Synthesis

**Figure 3:**
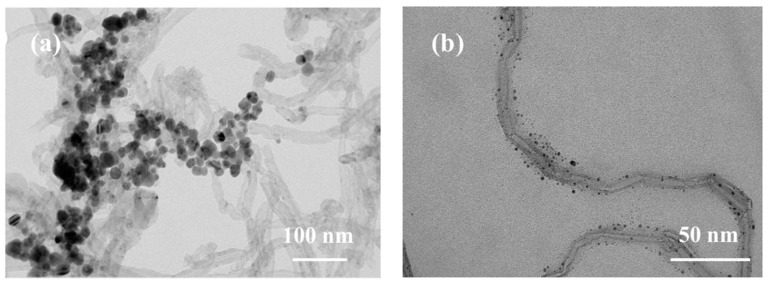
TEM images of Ag/MWNT composites synthesized with different dispersants. (**a**) SDS as dispersant; (**b**) SDBS as dispersant.

This chapter investigates the most effective method for synthesizing Ag/MWNT composites, comparing the effects of incorporating a modified Ag/MWNT solution with SDBS and SDS surfactants. Experimental results show that a dispersant at a 3:1 weight ratio between MWNTs and surfactant produces well-dispersed and stable MWNT nanofluids. As shown in Figure 3b, adding SDBS dispersant, followed by reduction with silver nitrate, ensures the effective dispersion of Ag/MWNTs in the solution. Figure 3a illustrates that adding SDS results in Ag NPs with an average size of about 10 nm or less. However, the Ag NPs show a wide range of morphologies, unevenly distributed across the MWNT surface, with high density. Notably, the Ag NPs do not appear to adsorb or interact directly with MWNTs, indicating the formation of a distinct micelle-like state.

In contrast, SDBS contains a benzene ring, and previous studies have investigated its interaction with the π-π bonds through molecular simulations [39]. This interaction forms a π-π bond between the benzene ring in SDBS and the graphite layer of MWNTs, enhancing MWNT dispersion. However, Ag NPs adsorb around MWNT instead of anchoring to its surface. This leads to reduced stability and anti-settling properties of Ag NPs. Over time, they undergo spontaneous agglomeration or detachment, further compromising stability. This phenomenon is due to the lack of adsorptive contact between Ag NPs and MWNTs, significantly reducing electrical conductivity. As a result, when used in a device, the composite material’s performance may be comparable to, or even worse than, that of MWNTs alone.

### 3.4. XPS Analysis and X-Ray Diffraction Analysis of Ag NPs Attachment

**Figure 4:**
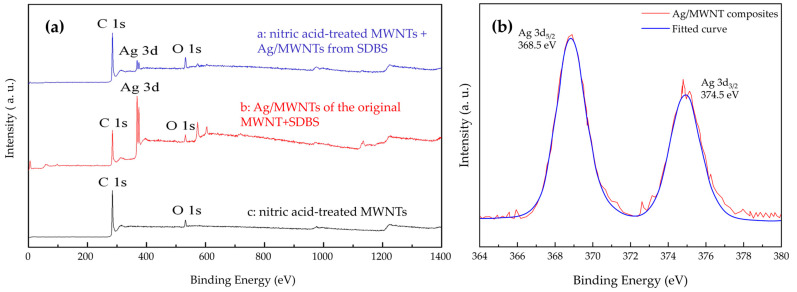
(**a**) XPS image of the sample, curve a: nitric acid-treated MWNTs + Ag/MWNTs from SDBS; curve b: Ag/MWNTs of the original MWNT+SDBS; curve c: nitric acid-treated MWNTs. (**b**) XPS images of Ag/MWNT composite samples.

XPS spectroscopy (X-ray: monochromatized AlKα, voltage 15 kV, current 10 mA) was used to investigate the surface properties of Ag/MWNTs, with the results shown in Figure 4. The measurements (c) showed an oxygen peak at 532 eV on the MWNT surface, indicating surface oxygen. This oxygen may arise from hydroxyl groups, water, or oxygen vacancies on the MWNT surface [40]. Regarding the Ag deposition, a peak at 368.5 eV corresponds to the Ag 3d5 phase. Peaks at 367.2 eV and 367.6 eV were attributed to Ag2+ and Ag+, respectively. Measurement (b) shows that the Ag 3d5 peak is 368.4 eV, matching the reported value for metallic Ag. This suggests that Ag+ was reduced to metallic Ag. The mass contents of C1s, Ag 3d, and O1s were found to be 66.63%, 27.93%, and 5.44%, respectively. The silver content (27.93%) to carboxylate content ratio on the MWNT surface is 1:5, indicating that the carboxylate groups fully adsorbed the Ag NPs.

Additionally, measurement results (a) show that the silver peaks of nitric acid-treated MWNTs are more pronounced than those of the direct mixing method, with Ag NPs adhesion reaching 14.97%, 82.1%, and 2.93%, respectively. These results suggest that nitric acid treatment significantly increased the surface functional group content on MWNTs, creating defects that provide more sites for silver attachment. As a result, Ag/MWNT composites with uniform surface attachment were formed.

**Figure 5:**
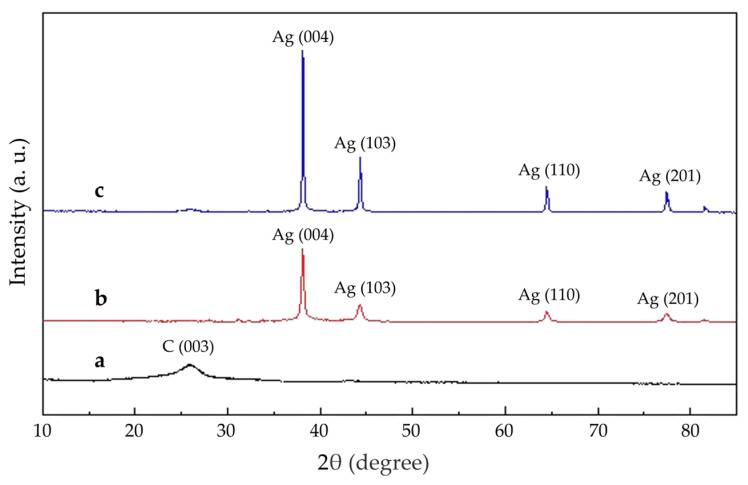
XRD image of the sample, (**a**) nitric acid-treated MWNTs + Ag/MWNTs from SDBS; (**b**) Ag/MWNTs of the original MWNT+SDBS; (**c**) nitric acid-treated MWNTs.

Figure 5 illustrates that the XRD patterns of Ag/MWNT show four distinct crystallographic planes: Ag(004), Ag(103), Ag(110), and Ag(201), corresponding to the (004), (103), (110), and (201) facets of MWNTs, respectively. The diffraction angles at 37.63°, 43.92°, 63.97°, and 77.34° [28] confirm the presence of these planes in the Ag/MWNT composites. The data shows a characteristic reflection peak at 26.4° for the MWNT sample, corresponding to the (002) facet of MWNT material [41]. This suggests that nitric acid treatment did not alter the surface structure of the MWNTs. Result b shows a sharp (004) peak, indicating optimal crystallization of Ag NPs and confirming their anchoring on the MWNT surface, which aligns with the transmission electron microscopy observations. Additionally, result c presents the XRD patterns of Ag/MWNT composites with negatively charged groups. The results reveal a more pronounced silver (004) peak on the nitric acid-treated MWNT surface. The negatively charged groups on the MWNT surface enhance the interaction between Ag NPs and the MWNT surface, resulting in a stronger attraction between Ag+ and MWNTs. This is consistent with the XPS results.

### 3.5. BET Analysis

BET (Brunauer–Emmett–Teller) surface area analysis was used to examine the properties of Ag/MWNT composites. Table 2 presents the BET surface area, total pore volume, and average pore diameter of Ag/MWNT samples synthesized using different methods. These results align with those previously reported by other researchers [42]. Table 2 shows that the nitric acid-treated MWNT sample B retains a large specific surface area, approaching the untreated MWNTs. This suggests that nitric acid treatment increases the functional group density on the MWNT surface. The BET surface areas of Ag/MWNT composites synthesized with a dispersant were 88.087 and 85.197 m^2^/g. The functionalized MWNTs with SDBS dispersant showed a more significant decrease in surface area. The BET surface area of Ag/MWNT composites decreased more noticeably than those with SDBS dispersant. This is likely due to the formation of micelles by the functionalized MWNTs, which interact with SDBS. These micelles seem to reduce the pores between the MWNTs. The BET surface area of Ag/MWNT composites made with nitric acid-treated MWNTs and the SDBS dispersant decreased. However, the reduction in pores suggests that the Ag/MWNT composites synthesized by this method were more uniformly dispersed, with Ag NPs adhering evenly to the surface of the carbon tubes. According to previous reports [40], when the BET surface area of Ag/MWNT composites is less than half that of the original MWNTs, the content of Ag NPs on the MWNT surface is higher.

**Figure 6:**
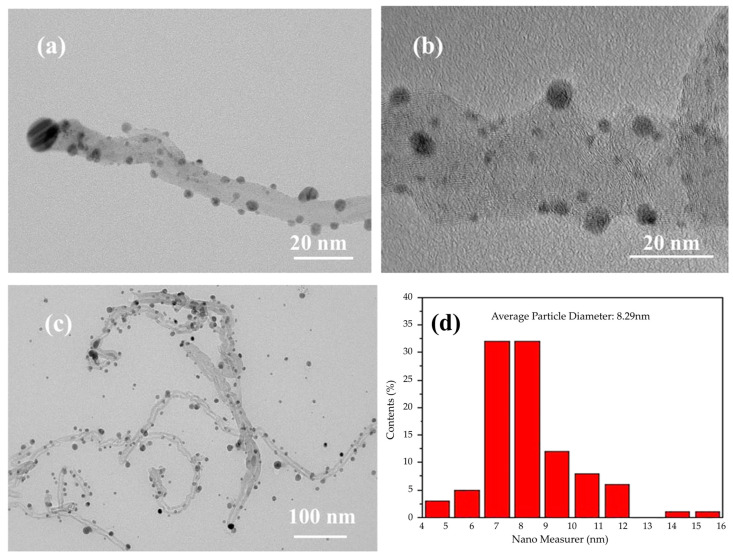
(**a**) shows the single TEM image of Ag/MWNT composite with SDBS added; (**b**) the localized TEM image of a single carbon nanotube; (**c**) the overall TEM image of Ag/MWNT composite with SDBS added; (**d**) histogram of Ag NPs particle size distribution in Ag/MWNT composites.

Figure 6a shows the morphology of Ag/MWNT composites synthesized by adding SDBS. A single TEM image of Ag/MWNT composites with added SDBS is shown in Figure 6b. The MWNT suspension was washed several times with ethanol to ensure that MWNTs did not form a micellar state with any residual dispersant. This process allowed for a more uniform deposition of Ag NPs on the surface of the MWNTs. Figure 6b presents a localized TEM image of a single MWNT within the Ag/MWNT composite containing SDBS. Figure 6c shows the overall TEM image of the Ag/MWNT composite with SDBS. Ag NPs are notably smaller and visible on the surface of the MWNTs. Ag NPs, having a consistent size and morphology, are uniformly distributed on the MWNT surface. The average size of the Ag NPs is approximately 8.76 nm, with a peak particle size around 7.8 nm.

The results showed that the optimal morphology of Ag/MWNT composites was achieved, with MWNTs exhibiting a binding state with Ag NPs. This observation suggests that the functional clusters on the MWNT surface act as an attraction, binding the Ag NPs to the MWNTs. Figure 6b shows the transmission electron microscopy results. The figure illustrates that Ag NPs are uniformly adsorbed on the MWNTs, with an average size of about 10 nm. The silver-coated MWNTs are approximately cylindrical, with the MWNTs as the inner layer and the silver plating as the outer layer. This configuration facilitates binding Ag NPs to each other, thereby establishing a conduction path in the electronic paste.

### 3.6. Effect of Ag/MWNTs on the Conductivity of Electronic Pastes

Researchers have reported using sheet silver powder as a base, supplemented with spherical silver powder that exhibits excellent electrical conductivity. The optimal performance of silver pastes was achieved when 60 wt.% of silver powder was added [28]. Incorporating an appropriate quantity of ultrafine powder has been shown to increase the overall surface area of the silver powder, thus reducing the thickness of the organic resin surrounding the silver powder particles. This, in turn, helps reduce the volume resistivity of the silver paste electrode after the curing process. The void ratio in the stacking structure was reduced by optimizing the size and ratio of ultrafine silver powder, improving the electrical conductivity of the silver paste. The performance of the silver pastes was also evaluated before and after introducing ultrafine powders.

The experimental slurry has a dosage of 20 g, with flaky silver powder (approximately 4 microns in particle size) as the primary filler. The initial sample differs only in the composition of the silver powder. In contrast, the next two samples (60 wt.%) are modified by altering the MWNT or Ag/MWNT composite material additive (secondary filler) in concentrations of 1–3%, respectively. Each experimental group consists of five samples, and the mean value is calculated to assess the effect of the MWNT or Ag/MWNT composite material on the slurry’s conductivity.

**Figure 7:**
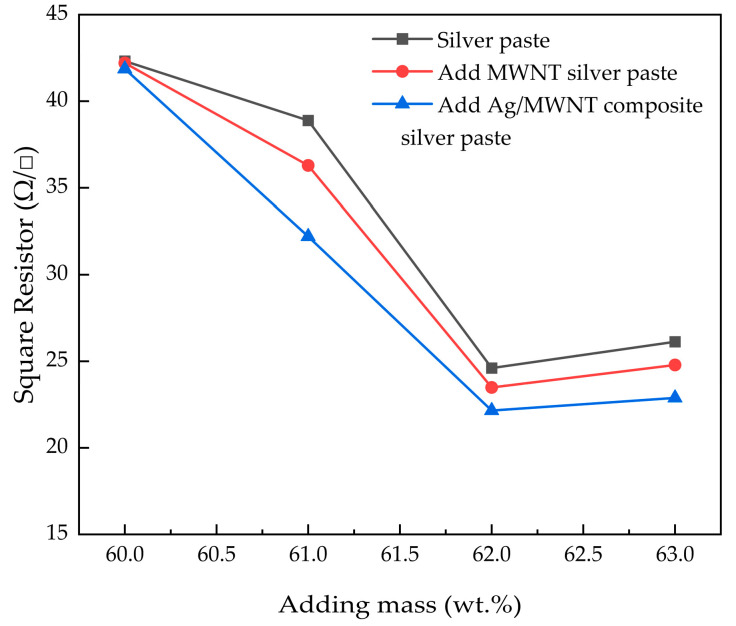
Comparison of electrical conductivity of different fillers Ag/MWNT/(Ag/MWNT).

As shown in Figure 7, incorporating MWNT and Ag/MWNT composites reduces the slurry’s resistance. Adding Ag/MWNT composites is more effective than adding MWNTs alone. This can be partly attributed to the role of MWNTs as effective conductors, which increases the total number of carriers in the system. Adding 3 wt.% Ag/MWNT composites led to a 32% reduction in square resistance compared to pure silver slurry. This improvement is due to the sintering bonding of Ag NPs on the MWNT surface and the interface connecting the silver flake powder. Additionally, the Ag NPs attached to MWNTs facilitate interfacial carrier transport. As a result, the square resistance was reduced by 32%, with sintering bonding occurring at the interface between Ag NPs on MWNTs and silver flake powder, enhancing carrier transport. The Ag NPs attached to MWNTs act as a bridge, connecting the MWNTs and silver flake powder to create multiple thermally conductive pathways, improving the interfacial transfer efficiency of carriers [43].

**Figure 8:**
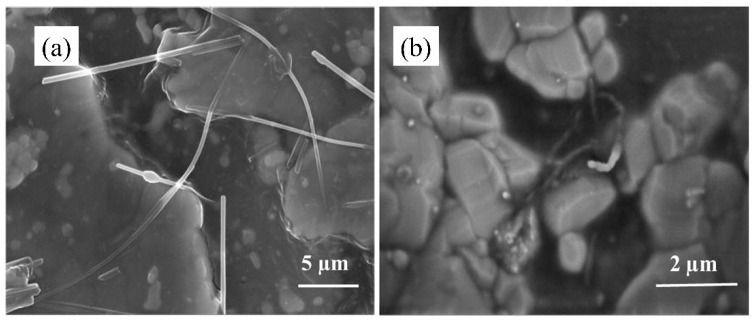
(**a**) SEM image of filled carbon nanotube e-paste, (**b**) SEM image of silver-modified carbon nanotube-filled paste.

The inherent challenges in wetting MWNTs with epoxy resin and its strong inter-tubular interactions cause it to agglomerate within the epoxy resin matrix. Figure 8 illustrates that nitric acid-treated MWNT surfaces exhibit functional groups such as -C=O, -OH, and -NH2, which improve wettability and promote uniform dispersion in slurry systems, preventing agglomeration. However, as shown in Figure 8a, the MWNTs and silver powder are in contact, resulting in significant inter-tube resistance. As a result, the electrical conductivity of the electronic paste shows only a minimal improvement in thermal conductivity. The slight conductivity enhancement can be attributed to the high conductivity of MWNTs, which increases the number of carriers in the system. Although the MWNTs form a conduction path between silver powder particles through simple contact, a significant interfacial transport barrier is created due to the high electrical resistance of the MWNT tube wall. This leads to a negligible enhancement in the slurry’s electrical conductivity, impacting the composite material’s overall conductivity. After incorporating the Ag/MWNT composites, as shown in Figure 8b, sintering bonding was observed at the interface between the Ag NPs on the MWNT surface and the flaky silver powder.

The Ag NPs on the surface of the MWNTs reduce the van der Waals forces between MWNTs, significantly enhancing their dispersion and wetting in the polymer matrix. This improvement leads to enhanced electrical and thermal conductivity in the electronic paste. The Ag NPs, with their high sintering activity, can bond with the silver particles in the paste, forming seamless metal-to-metal contacts. Additionally, the sintering of Ag NPs on the silver-modified MWNTs with other silver particles establishes multiple conduction paths between the flaky silver powders, further improving the electrical and thermal conductivity of the epoxy resin-based electronic paste.

### 3.7. Effect of Ag/MWNTs on the Thermal Resistance of Electronic Pastes

**Figure 9:**
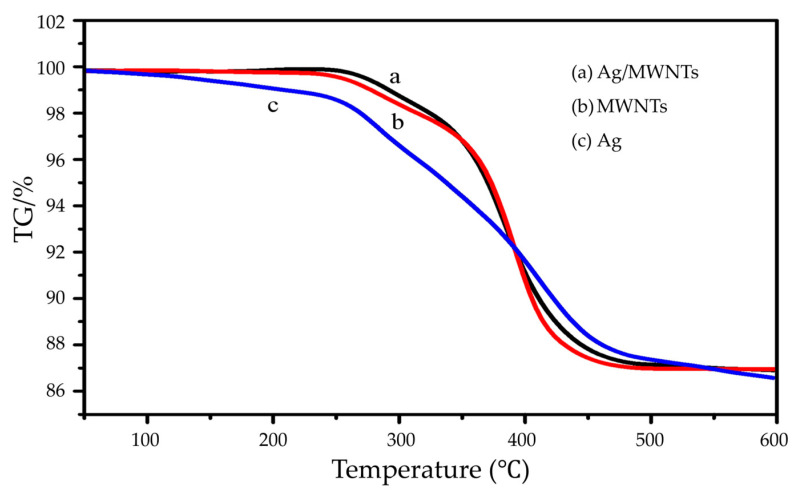
Comparison of TG analysis of different fillers.

The maximum thermal decomposition temperature corresponding to the weight loss of 5 wt.%, 10 wt.%, and the peak of the heat loss curve (DTG) of the sintered samples is a commonly used characteristic thermal stability parameter, which can reflect the thermal aging performance of the resin polymers and their composites (Figure 9 (a): silver paste with Ag/MWNTs added; b: silver paste with MWNTs added; c: conventional silver paste). The TGA curves of the MWNT/epoxy resin composites in the presence of silver paste (Figure 9 (a–c)) demonstrate a clear trend of increasing decomposition temperature with increasing amounts of MWNT filling. This indicates that adding functional fillers with excellent thermal stability in organic resins can effectively enhance the heat resistance of electronic pastes [44]. The results suggest that the incorporation of MWNTs into epoxy resin can improve its thermal stability, suggesting a potential for enhanced heat resistance in electronic pastes. Functionalized MWNTs have been shown to enhance compatibility with the epoxy resin matrix through surface modification. However, it is challenging to be wetted by the epoxy resin itself, and strong interactions between the tubes are prone to agglomeration, forming defects in the paste. These defects can lead to the thermal decomposition of the epoxy resin [45].

Compared to MWNTs, Ag/MWNT composites exhibit higher heat resistance than silver paste—the addition of 3 wt.% Ag/MWNT composites will increase the transition temperature of the paste from 273 °C to 312 °C for pure silver paste, which is higher than the corresponding transition temperature of 307 °C for MWNTs with the same filling amount. This outcome suggests that the Ag modification of MWNTs restricts the thermal vibration of the C-C bonds of the epoxy resin matrix chain segments. Additionally, the silver modification of MWNT binds more epoxy resin chain segments on its surface, which increases the composites’ thermal stability.

Additionally, when Ag NPs on the surface of the MWNTs absorb heat, they rapidly transfer this heat to their attached MWNTs, which function as thermal conductors with exceptionally high thermal conductivity. This facilitates the swift transfer of the absorbed external energy, thereby mitigating the thermal decomposition process of the Ag/MWNT pastes [31]. The collective impact of these factors has culminated in a substantial enhancement in the thermal stability of slurries comprising Ag/MWNT composites.

### 3.8. Ag/MWNT Effect on Thermal Conductivity of Electronic Pastes

**Figure 10:**
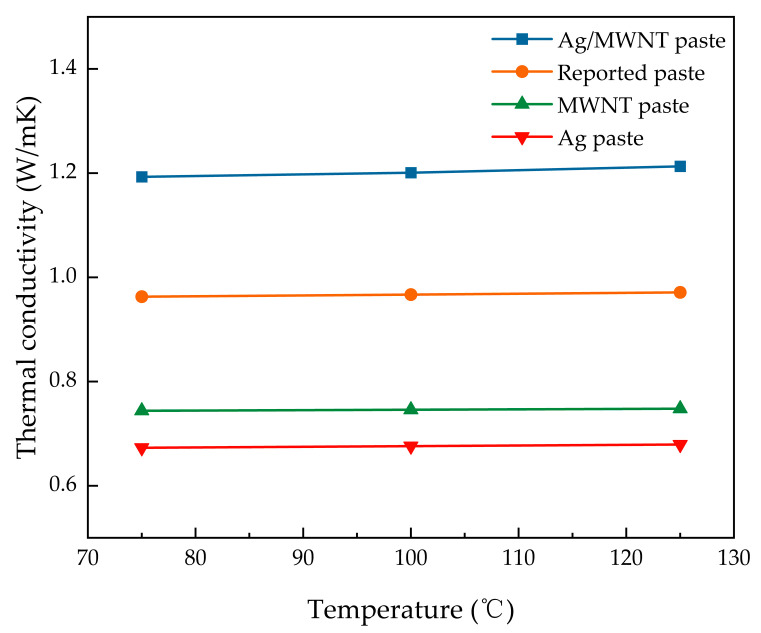
Comparison of thermal conductivity of different pastes.

Figure 10 illustrates the thermal conductivity of electronic pastes with different materials incorporated. At a 3 wt.% fill rate, adding MWNTs increased the thermal conductivity of the paste from 0.673 W/m-K to 0.744 W/m-K. Incorporating Ag/MWNT composites into the epoxy resin boosted the thermal conductivity to 1.202 W/m-K, representing an increase of 61.5% and 78.6%, respectively, compared to silver paste. Compared to the reported values for electronic pastes (0.963 W/m-K), this represents a 24.8% improvement [28]. This enhancement is attributed to the high sintering activity of silver particles in the composite, allowing them to form seamless metal-to-metal contacts. The MWNTs within the silver powder contribute significantly to forming numerous thermal conductivity pathways, resulting in a composite with high thermal conductivity.

However, the high silver content in the Ag/Epoxy system leads to brittleness. Incorporating silver-modified MWNTs improves the system’s mechanical strength and thermal stability [27,46]. The mismatch in thermal expansion coefficients between the MWNTs and the resin matrix initially enhances thermal conductivity, but this effect diminishes at higher temperatures. This reduction is due to decreased connectivity between the MWNTs and the filler, resulting in lower material densification and increased phonon scattering at the filler interfaces. Nevertheless, incorporating 3 wt.% Ag/MWNT composites increase thermal conductivity by 43%, improving thermal conduction between the chip and the heat sink.

The synthesis of Ag/MWNT composites through surface modification results in Ag NPs on the MWNT surface. This configuration reduces the van der Waals forces between MWNTs, improving their dispersion in the matrix while preserving the low-melting-point sintering activity, allowing for metal-to-metal bonding. This structure provides the composites with metal-like characteristics [27]. The enhanced thermal conductivity of Ag/MWNT composites, surpassing that of single fillers, expands the potential applications of electronic pastes, making them more versatile in various technological settings.

## 4. Conclusions

This paper focuses on synthesizing Ag/MWNT composites and studying the impact of functional additives in electronic pastes on their thermal conductivity. The surface and cross-sectional morphology, thermal conductivity, electrical conductivity, and thixotropic index of the low-temperature-cured electronic pastes were characterized and measured as follows:

(1) The addition of the dispersant SDBS, combined with the weak reducing agent ascorbic acid, facilitates the reduction of Ag NPs to an average size of 10 nm on the MWNT surface, achieving an Ag loading of 80 wt.% on the MWNTs. This method effectively controls the growth of Ag NPs, maintaining optimal particle size for good sintering activity. Additionally, the high specific surface area of MWNTs and the heat resistance of the thermal interface material are fully utilized.
(2) In the electronic paste containing Ag/MWNT composites, MWNT surface Ag NPs and silver powder are sintered together, significantly enhancing carrier transport efficiency. This compensates for the lack of thermal conductivity in a single filler. The sintered structure of the composites, combined with the introduction of MWNTs, enhances the system’s mechanical strength and thermal stability while reducing the mismatch between the thermal expansion coefficients of MWNTs and the resin. The integration of MWNTs improves mechanical strength and thermal stability while mitigating the mismatch between the thermal expansion coefficients of MWNTs and the resin. Additionally, the sintered connection between Ag NPs on the MWNT surface and silver particles reduces the carrier transport barrier, significantly improving the transmission efficiency of phonons and electrons at the filler interface. This leads to a 32.1% improvement in thermal conductivity and a 43.1% improvement in the electrical conductivity of the electronic pastes.

## Figures and Tables

**Table 1:** Experimental reagents.

Reagent Name	Reagent type	Manufacturer
Silver nitrate (AgNO_3_)	AR, 99%	Xilong Scientific Co., Ltd. Shenzhen, China.
L-ascorbic acid (C_6_H_8_O_6_)	AR, 99%	Xilong Scientific Co., Ltd.
Hydrazine Hydrate (N_2_H_4_·H_2_O)	AR, 99%	Xilong Scientific Co., Ltd.
Anhydrous Ethanol (C_2_H_5_OH)	AR, 99%	Xilong Scientific Co., Ltd.
Epoxy Resin (chemistry)	CP	Xilong Scientific Co., Ltd.
Flake Silver Powder	99.5%	Kunming Precious Metals Institute. Kunming, China.
Silicon Dioxide (SiO_2_)	CP	Xilong Scientific Co., Ltd.
Multi-Walled Carbon Nanotubes	99.9%	Guangxi Qinglu New Materials Technology Co., Ltd. Nanning, China.
Sodium Dodecyl Sulfate (SDS)	AR, 99%	Xilong Scientific Co., Ltd.
Sodium Dodecylbenzene Sulfonate (SDBS)	AR, 99%	Xilong Scientific Co., Ltd.
Formaldehyde (HCHO)	AR, 99%	Guangxi Qinglu New Materials Technology Co., Ltd. Liuzhou, China.

**Table 2:** BET analysis results.

Numbers	BET Surface Area (m^2^/g)	Pore Volume (mL/g)	Pore Diameter (nm)
A. Unprocessed MWNT	172.743	0.669	15.51
Nitric acid treatment of MWNT	168.741	0.879	20.60
Nitric acid treatment of MWNT + SDS with Ag/MWNT	88.197	0.461	21.69
Nitric acid treatment of MWNT + SDBS with Ag/MWNT	84.087	0.447	20.32

## Data Availability

The datasets generated during and/or analyzed during the current study are available from the corresponding author on reasonable request.

## Abbreviations

The following abbreviations are used in this manuscript:

Ag NPs	Silver nanoparticles
MWNTs	Multi-walled carbon nanotubes
Ag/MWNTs	Silver nanoparticle-coated multi-walled carbon nanotubes

## References

[1] Liu M., Hou Z., Huang B., Gou L., Zhang P. (2017). The preparation, characterization, and properties of silver nanoparticle reinforced reduced graphene oxide–poly(amidoamine) nanocomposites. J. Appl. Polym. Sci..

[2] Shahil K.M.F., Balandin A.A. (2012). Graphene–Multilayer Graphene Nanocomposites as Highly Efficient Thermal Interface Materials. Nano Lett..

[3] Sun X., Yu A., Ramesh P., Bekyarova E., Itkis M.E., Haddon R.C. (2011). Oxidized Graphite Nanoplatelets as an Improved Filler for Thermally Conducting Epoxy-Matrix Composites. J. Electron. Packag..

[4] Chen L., Zhao P., Xie H., Yu W. (2016). Thermal properties of epoxy resin based thermal interfacial materials by filling Ag nanoparticle-decorated graphene nanosheets. Compos. Sci. Technol..

[5] Goyal V., Balandin A.A. (2012). Thermal properties of the hybrid graphene-metal nano-micro-composites: Applications in thermal interface materials. Appl. Phys. Lett..

[6] Shen X., Jiang C., Li Y., Huang J. (2016). Thermal metamaterial for convergent transfer of conductive heat with high efficiency. Appl. Phys. Lett..

[7] Yuan W., Xiao Q., Li L., Xu T. (2016). Thermal conductivity of epoxy adhesive enhanced by hybrid graphene oxide/AlN particles. Appl. Therm. Eng..

[8] Li Y., Wu Y., Ong B.S. (2005). Facile Synthesis of Silver Nanoparticles Useful for Fabrication of High-Conductivity Elements for Printed Electronics. J. Am. Chem. Soc..

[9] Kul E., Aladağ L.O., Yesildal R. (2016). Evaluation of thermal conductivity and flexural strength properties of poly(methyl methacrylate) denture base material reinforced with different fillers. J. Prosthet. Dent..

[10] Yu F., Cui J., Zhou Z., Fang K., Johnson R.W., Hamilton M.C. (2017). Reliability of Ag Sintering for Power Semiconductor Die Attach in High-Temperature Applications. IEEE Trans. Power Electron..

[11] Gao Y., Marconnet A.M., Xiang R., Maruyama S., Goodson K.E. (2013). Heat Capacity, Thermal Conductivity, and Interface Resistance Extraction for Single-Walled Carbon Nanotube Films Using Frequency-Domain Thermoreflectance. IEEE Trans. Compon. Packag. Manuf. Technol..

[12] Paknejad S.A., Mannan S.H. (2017). Review of silver nanoparticle based die attach materials for high power/temperature applications. Microelectron. Reliab..

[13] Zhou Y., Luo Z., Zhuang X., Liu F. (2016). Multilayer-structured high-performance nanocomposites based on a combination of silver nanoparticles and nanowires. Mater. Lett..

[14] Seo M., Kim J.S., Lee J.G., Kim S.B., Koo S.M. (2016). The effect of silver particle size and organic stabilizers on the conductivity of silver particulate films in thermal sintering processes. Thin Solid Films.

[15] Zhao Y., Mumby-Croft P., Jones S., Dai A., Dou Z., Wang Y., Qin F. Silver sintering die attach process for IGBT power module production. Proceedings of the 2017 IEEE Applied Power Electronics Conference and Exposition (APEC).

[16] Rivière L., Lonjon A., Dantras E., Lacabanne C., Olivier P., Gleizes N.R. (2016). Silver fillers aspect ratio influence on electrical and thermal conductivity in PEEK/Ag nanocomposites. Eur. Polym. J..

[17] Ma A.J., Wu Y.L., Chen W.X., Wu X. (2014). Preparation and Properties of Multi-Walled Carbon Nanotubes/Carbon Fiber/Epoxy Composites. Polym. Compos..

[18] Guadagno L., Lamberti P., Tucci V., Vertuccio L. (2021). Self-Sensing Nanocomposites for Structural Applications: Choice Criteria. Nanomaterials.

[19] Aman Qazi R., Saleem Khan M., Siddiq M., Ullah R., Shah L.A., Ali M. (2020). Synthesis and characterization of functionalized MWCNTs/PMMA composites: Device fabrication for RH sensing. Polym. Technol. Mater..

[20] Wang H., Guo J., Li J., Wei J. (2011). Fabrication of bimetallic nanoparticles/multi-walled carbon nanotubes composites for microelectronic circuits. Carbon.

[21] Nagajyothi P.C., Veeranjaneya Reddy L., Devarayapalli K.C., Vattikuti S.V.P., Wee Y.J., Shim J. (2021). Environmentally Friendly Synthesis: Photocatalytic Dye Degradation and Bacteria Inactivation Using Ag/f-MWCNTs Composite. J. Clust. Sci..

[22] Hussain T., Jabeen S., Shehzad K., Mujahid A., Ahmad M.N., Farooqi Z.H., Raza M.H. (2018). Polyaniline/Silver Decorated-MWCNT Composites With Enhanced Electrical and Thermal Properties. Polym. Compos..

[23] Liu C., Gan G., Li Y., Yu X., Cheng J., Tang L. (2022). Preparation of Silver-Epoxy Resin Paste with Ag/MWCNTs Composites Using Lodinated Multi-walled Carbon Nanotubes. Rare Met. Mater. Eng..

[24] Dinh N.X., Van Quy N., Huy T.Q., Le A.-T. (2015). Decoration of Silver Nanoparticles on Multiwalled Carbon Nanotubes: Antibacterial Mechanism and Ultrastructural Analysis. J. Nanomater..

[25] Bi C., Zhao D. (2018). Enhancement in mechanical properties and conductivity of modified epoxy resin composites. High Perform. Polym..

[26] Yadav A., Upadhyaya A., Gope J., Gupta S.K., Negi C.M.S. (2020). Silver-decorated multiwall carbon nanotubes: Synthesis characterization and application in polymer composite-based devices. J. Mater. Sci. Mater. Electron..

[27] Pal H., Sharma V. (2015). Thermal conductivity of carbon nanotube–silver composite. Trans. Nonferrous Met. Soc. China.

[28] Li Y., Gan G., Huang Y., Yu X., Cheng J., Liu C. (2019). Ag-NPs/MWCNT composite-modified silver-epoxy paste with improved thermal conductivity. RSC Adv..

[29] Wang Y., Jing D., Xiong Z., Hu Y., Li W., Wu H., Zuo C. (2024). Ag-MWCNT Composites for Improving the Electrical and Thermal Properties of Electronic Paste. Polymers.

[30] Natsuki J., Natsuki T. (2023). Silver Nanoparticle/Carbon Nanotube Hybrid Nanocomposites: One-Step Green Synthesis, Properties, and Applications. Nanomaterials.

[31] Suh D., Moon C.M., Kim D., Baik S. (2016). Ultrahigh Thermal Conductivity of Interface Materials by Silver-Functionalized Carbon Nanotube Phonon Conduits. Adv Mater..

[32] Amoli B.M., Gumfekar S., Hu A., Zhou Y.N., Zhao B. (2012). Thiocarboxylate functionalization of silver nanoparticles: Effect of chain length on the electrical conductivity of nanoparticles and their polymer composites. J. Mater. Chem..

[33] Meschi Amoli B., Hu A., Zhou N.Y., Zhao B. (2015). Recent progresses on hybrid micro–nano filler systems for electrically conductive adhesives (ECAs) applications. J. Mater. Sci. Mater. Electron..

[34] Daoush W.M., Hong S.H. (2013). Synthesis of multi-walled carbon nanotube/silver nanocomposite powders by chemical reduction in aqueous solution. J. Exp. Nanosci..

[35] Zhao Q., Xie M., Liu Y., Yi J. (2017). Improved electroless plating method through ultrasonic spray atomization for depositing silver nanoparticles on multi-walled carbon nanotubes. Appl. Surf. Sci..

[36] Rodríguez-Galván A., Contreras-Torres F.F., Basiuk E.V., Heredia A., Basiuk V.A. (2013). Deposition of silver nanoparticles onto human serum albumin-functionalised multi-walled carbon nanotubes. Can. J. Chem. Eng..

[37] Korusenko P.M., Knyazev E.V., Vinogradov A.S., Kharisova K.A., Filippova S.S., Rodionova U.M., Levin O.V., Alekseeva E.V. (2024). Structure and Electrocatalytic Properties of Sulfur-Containing Multi-Walled Carbon Nanotubes on a Titanium Substrate Modified by a Helium Ion Beam. Nanomaterials.

[38] Zhao W., Wang H., Qin X., Wang X., Zhao Z., Miao Z., Chen L., Shan M., Fang Y., Chen Q. (2009). A novel nonenzymatic hydrogen peroxide sensor based on multi-wall carbon nanotube/silver nanoparticle nanohybrids modified gold electrode. Talanta.

[39] Matsuura Y. (2025). Coherent electron transport through multiple π-π stacked benzene rings. Comput. Theor. Chem..

[40] Chen L., Xie H., Yu W. (2012). Multi-walled carbon nanotube/silver nanoparticles used for thermal transportation. J. Mater. Sci..

[41] Yalovega G.E., Brzhezinskaya M., Dmitriev V.O., Shmatko V.A., Ershov I.V., Ulyankina A.A., Chernysheva D.V., Smirnova N.V. (2024). Interfacial Interaction in MeO_x_/MWNTs (Me–Cu, Ni) Nanostructures as Efficient Electrode Materials for High-Performance Supercapacitors. Nanomaterials.

[42] Peigney A., Laurent C., Flahaut E., Bacsa R., Rousset A. (2001). Specific surface area of carbon nanotubes and bundles of carbon nanotubes. Carbon.

[43] Yusof Y., Zaidi M.I., Johan M.R. (2016). Enhanced Structural, Thermal, and Electrical Properties of Multiwalled Carbon Nanotubes Hybridized with Silver Nanoparticles. J. Nanomater..

[44] Vilčáková J., Moučka R., Svoboda P., Ilčíková M., Kazantseva N., Hřibová M., Mičušík M., Omastová M. (2012). Effect of Surfactants and Manufacturing Methods on the Electrical and Thermal Conductivity of Carbon Nanotube/Silicone Composites. Molecules.

[45] Trinidad J., Amoli B.M., Zhang W., Pal R., Zhao B. (2016). Effect of SDS decoration of graphene on the rheological and electrical properties of graphene-filled epoxy/Ag composites. J. Mater. Sci. Mater. Electron..

[46] Choi J., Rhee K., Park S. (2015). Influence of electrolessly silver-plated multi-walled carbon nanotubes on thermal conductivity of epoxy matrix nanocomposites. Compos. Part B Eng..

